# Prone Positioning for Management of Fat Embolism Syndrome in a Patient with Spine Fracture; An Unusual Scenario and Review of Literature

**DOI:** 10.29252/beat-070217

**Published:** 2019-04

**Authors:** Abhay Tyagi, Richa Aggarwal, Kapil dev Soni, Anjan Trikha

**Affiliations:** 1 *Department of Anesthesiology, Critical Care and Pain Medicine, All India Institute of Medical Sciences, New Delhi, India*; 2 *Department of Critical Care, JPNATC, All India Institute of Medical Sciences, New Delhi, India*

**Keywords:** Fat embolism, Prone position, Trauma, ARDS

## Abstract

Fat embolism syndrome is a rare but fatal complication seen commonly in patients with polytrauma. Its earliest manifestation is hypoxemia due to deposition of fat globules in pulmonary circulation which can progress to severe acute respiratory distress syndrome, the treatment of which is mainly supportive. We describe the case of a 17-year-old male who was admitted in our intensive care unit (ICU) for severe hypoxemia due to fat embolism. He had burst fracture of 5th lumbar vertebra with canal compromise along with other fractures. Failing conventional ventilation, the patient was placed in prone position taking proper precautions in positioning giving due consideration to his unstable lumbar spine. There was no neurological insult and in the next two days, he was weaned off from the ventilator. Though prone position is relatively contraindicated in patients with unstable spine, we employed early prone positioning taking adequate precautions, the benefit of which we believe outweighed the risk.

## Introduction

Fat embolism, described as the presence of fat globules in pulmonary and systemic circulation is commonly seen in patients with long bone fracture and other major trauma [1]. Fat embolism syndrome (FES) however, is a rare but potentially lethal complication with respiratory and neurological changes along with characteristic petechial rash [2]. This triad of FES however, is seen rarely together with petechial rash seen in only 50-60% cases only [3]. Atypical presentations are common making FES a diagnostic enigma for clinicians. Respiratory manifestation of FES is the earliest to appear typically within 24-72 h characterized by dyspnea, tachypnea and hypoxemia with chest radiograph showing fluffy lung infiltrates [4]. The severity of these symptoms vary from mild to fulminant and can progress to severe acute respiratory distress syndrome (ARDS). The application of low tidal volume ventilation and positive end expiratory pressure for improving oxygenation in these patients is now a clinical standard [5,6]. 

Significant benefits of early proning in severe ARDS have recently been described in literature [7] and is being used increasingly in clinical practice nowadays. However, presence of unstable spine fracture and pelvic fracture are thought to be as contraindications to prone positioning [5,7]. We here in describe a case of an atypical FES with unstable lumbar spine fracture where early prone positioning was employed for severe ARDS resulting in dramatic recovery of the patient. 

## Case Presentation

A 17-year-old boy with history of fall from height of approximately 15 meters presented to our institution after receiving primary care at another hospital. At presentation, he was conscious, hemodynamically stable maintaining oxygen saturation at 98% on room air with no visible signs of respiratory distress. His Glasgow coma scale (GCS) was 15, and was able to move all four limbs.  The patient had sustained open fracture both bone left leg along with fracture right ankle. 

**Fig. 1:**
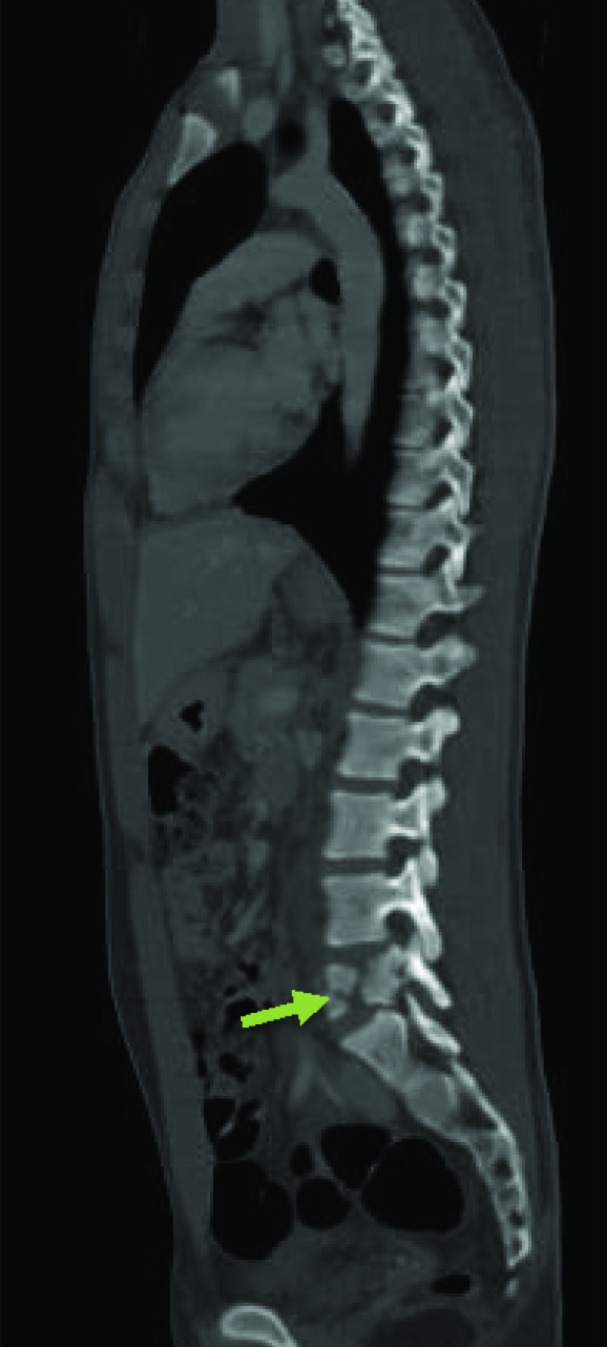
Sagittal Computed Tomography (CT) scan of the whole spine showing burst fracture at L5 (arrow) with canal compromise (Magerl A3)

Chest radiograph showed no intrathoracic injury with normal lung parenchyma. Computed Tomography showed burst fracture of fifth lumbar vertebra with canal compromise (Figure 1) and ruled out any injury to head, cervical spine, thorax and abdomen. After primary care, patient was admitted to the orthopedic ward for spine stabilization surgery and surgery for lower limb fracture.

**Fig. 2:**
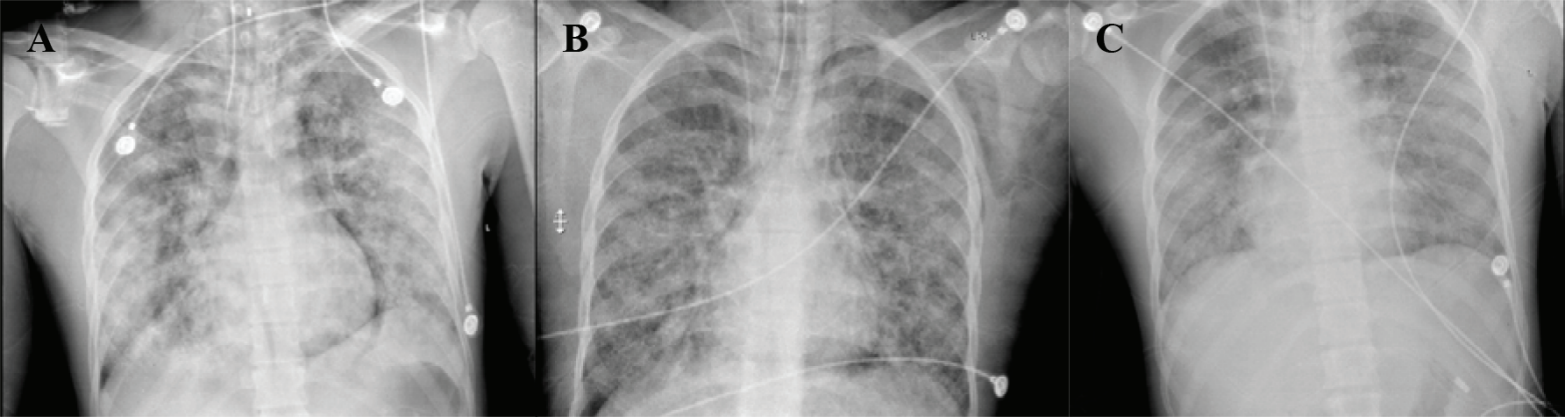
Serial images of daily chest radiographs taken during patient’s stay at the ICU. A) image on day 1st;B) image on day 2ndafter 16 hours of prone positioning; C) image on day 3

On day four post-admission, an emergency consultation call was sent to our intensive care unit (ICU) in view of patient’s deteriorating status. When seen, he was grossly pale and febrile at 101 F, pulse rate of 140 per minute, systolic blood pressure of 80 mmHg and respiratory rate of 32 per minute maintaining oxygen saturation around 90% on oxygen face-mask. Patient’s GCS was 15. Chest auscultation revealed bilateral diffuse coarse crepitation and he was immediately transferred to the ICU. Initial arterial blood gas (ABG) showed partial pressure oxygen (pO2) of 49mm Hg on oxygen by face mask. Patient was intubated, sedated, paralyzed and put on mechanical ventilation with initial settings of volume assist control and high positive end expiratory pressure (PEEP). Central venous catheter was secured in right internal jugular vein under ultrasound guidance. An arterial line was secured in right radial artery for invasive blood pressure and arterial blood gas analysis. Chest radiograph showed bilateral fluffy opacities (Figure 2). Preliminary blood investigations were mostly unremarkable except for hemoglobin of 6.8 mg/dl and raised ESR of 44. Fundus exam specific for FES was normal and there was no petechial rash on general examination. Urine for fat globules was positive. Over the next few hours, patient’s hypoxemia worsened requiring higher fraction inhaled oxygen (FiO2) of up to 0.8 and PEEP of 16 cm water. A decision was made to turn the patient into prone position after discussion with the orthopedic surgery team in view of unstable lumbar spine fracture. Patient’s family were informed about the specific risks and benefits of prone positioning, particularly in a patient with pre-existing unstable lumbar spine fracture and a written consent for the same was obtained. Positioning was done with the help of 5 trained ICU staff, using logrolling technique for turning the patient lateral followed by a 6th member placing the spinal board under the patient.  After securing the patient on the spinal board, he was then shifted to one edge of the bed while the head gel support, chest and pelvic roll were placed in position. Patient was then shifted to prone position and spinal board was removed. Patient’s hands were abducted and placed next to the head and all the pressure points were padded using pillows and cotton rolls.

 Over the next 16 hours, we kept our patient sedated and paralyzed in prone position during which he received targeted fluid therapy and remained hemodynamically stable. Wake up test and pupil examination were done at regular intervals. Serial ABG’s showed dramatic improvement with Fio2 requirement decreasing to 0.4 and PEEP of 8 following which patient was repositioned supine. Two units packed red cells were transfused while the patient was prone. Chest radiograph showed resolving lung infiltrates and we decided to electively mechanically ventilate our patient for next 24 hours in supine position. Weaning was started next day and patient was extubated a day after and transferred back to ward 24 hours later. 

The patient later on went on to have multiple surgical procedures for injuries to spine and bilateral lower limbs. He was then followed for 8 weeks in the out-patient department following discharge during which he recovered well without any neurovascular deficit.

## Discussion

The presence of fat globules in pulmonary and systemic circulation is most commonly seen after traumatic long bone fractures but has also been reported in multiple clinical conditions such as pancreatitis, fatty liver and procedures like bone marrow transplantation, liposuction and intramedullary nailing [2]. However, only few patients with fat embolism progress to the serious complication of fat embolism syndrome suggesting involvement of additional factors in development of FES. The widely accepted biochemical theory suggests that break-down of circulating fat emboli by lipoprotein lipase releases free fatty acids and other toxic metabolites into the pulmonary circulation causing endothelial damage and increased vascular permeability ultimately resulting in an ARDS like picture in these patients [2].

The clinical presentation of FES is variable and diagnostic criteria described in literature are helpful to some extent but their reliability remains questionable and a strong clinical suspicion is imperative for diagnosis. We describe a relatively atypical presentation of Fat embolism syndrome secondary to an unfixed open type III tibial fracture occurring five days after the injury. In our case, with the exception of a striking chest radiograph showing bilateral infiltrates in snowstorm appearance explaining for pronounced hypoxemia and tachypnea, the characteristic petechial rash was absent and neurological examination remained unremarkable in our patient. Rest of the biochemical investigations specific for FES were also normal. In view of patient’s fever and raised total leukocyte count, blood, urine and broncho-alveolar lavage samples for culture were sent which turned out to be negative. Serum procalcitonin was equivocal for any infectious etiology. The diagnosis of FES was made on the basis of Gurd’s criteria [8] with respiratory insufficiency and signs of pulmonary edema serving as the major criteria while fever, tachycardia, raised ESR and anaemia were minor criteria.

The treatment of FES is mainly supportive. The initial presentation in terms of hypoxemia and respiratory rate of 35/min at the time of ICU admission mandated endotracheal intubation and mechanical ventilation with application of PEEP in our patient. However, with worsening hypoxemia requiring FiO2 of more than 0.8 within few hours of ICU admission made us to employ prone positioning in our patient.

 Prone positioning has been employed to improve oxygenation in patients with ARDS with mortality benefit reported by meta-analyses and multiple randomized controlled studies [9,10]. The rationale behind prone positioning being that it helps in homogenization of ventilation by increasing alveolar recruitment, thereby improving oxygenation. By preventing over-inflation of certain lung regions, it also prevents ventilator induced lung injury in patients. Benefits of early prone positioning in patients with severe ARDS (P/F ratio <150) were demonstrated in the landmark study by Guerin *et al*., [7] in PROSEVA study group which showed 60% reduction in mortality(*p*<0.001) in patients with severe ARDS who were ventilated in prone position compared to those who were ventilated in supine position. The concept of early proning is now well accepted. The technical aspects of prone positioning however, are challenging and can have adverse effects of their own such as endotracheal tube mal-positioning, increased intraocular pressure, nerve injuries and pressure injuries [11]. 

Since the presentation of severe FES is similar to that of severe ARDS like in our patient, prone positioning was justified to improve failing oxygenation in our patient. However, in the review of studies employing prone positioning in patients with ARDS, a common contra-indication for prone position has been reported, i.e., presence of unstable spine and fracture pelvis. In our case, our patient had sustained fracture of 5th lumbar vertebra with canal compromise which made our case challenging. However, in the presence of skilled nursing and experienced staff well versed with technical details and complications of prone positioning, we had the motivation to employ early prone positioning while mitigating the risk associated in changing position with an unstable lumbar spine. This resulted in faster recovery in our patient with no adverse effects.

 While reviewing literature, we found a case report describing a patient with cerebral and pulmonary fat embolism in ARDS who suffered brain death secondary to acute tonsillar herniation leaving the authors to question the safety of prone positioning and low tidal volume ventilation in patients with cerebral fat embolism [12]. In our patient, there was no cerebral involvement from presentation to recovery. Despite the fact, we monitored our patient with pupil examination and wake up test at frequent intervals while he was prone keeping in mind the grave consequences of cerebral fat embolism that could have met us while managing our patient [13,14].

The use of corticosteroids for prevention of FES has been controversial with only a few small sample sized randomized studies supporting their use. Bederman *et al*., [15] in their review and meta-analysis concluded that corticosteroids could prevent the development of FES and hypoxia but do not affect mortality outcome. At the same time, due to the presence of small sample sized studies with weak methodologies, they advised caution in interpretation of their results. 

However, once the FES has set in, there is no role of corticosteroids in its treatment and even can be detrimental to the overall outcome, given their propensity to decrease immunity and cause peptic ulcer disease which severely ill patients in Trauma ICU are already predisposed to. As an institutional policy we do not prophylactically administer corticosteroids regularly in our trauma patients.

This patient later on went on to have multiple surgical procedures such as spinal instrumentation, open reduction and fixation with plating for right syndesmotic ankle fracture and external fixation for right lower leg both bone fracture. He was then followed for 8 weeks in the out-patient department following discharge during which he recovered well without any neurovascular deficit. 

In conclusion, fat embolism syndrome remains a clinical diagnosis and should be strongly suspected in polytrauma patients developing respiratory distress. Early prone positioning in patients with ARDS helps treat refractory hypoxemia and can be life-saving. The relative contra-indication of unstable spine should not preclude in prone positioning of patients in severe ARDS provided a skilled staff well versed with practicalities of prone positioning is present.

## Conflict of interest:

The authors declare there is no conflict of interest
